# Editorial: New cerebrospinal fluid research to uncover mechanisms driving neurological and psychiatric diseases, volume II

**DOI:** 10.3389/fneur.2023.1346377

**Published:** 2023-12-12

**Authors:** Thomas Skripuletz, Øivind Torkildsen

**Affiliations:** ^1^Department of Neurology, Hannover Medical School, Hannover, Germany; ^2^Department of Neurology, Neuro-SysMed, Haukeland University Hospital, Bergen, Norway; ^3^Department of Clinical Medicine, University of Bergen, Bergen, Norway

**Keywords:** CSF, CNS, neurology, psychiatry, fluid markers

Cerebrospinal fluid (CSF) examination is a key procedure in diagnosis of autoimmune, infectious and malignant diseases of the nervous system. In the last years, the relevance of CSF analysis in neurodegenerative and neuropsychiatric disorders further increased. Since the discovery of autoimmune encephalitis (AE) driven by autoantibodies directed to the N-methyl-D-aspartate (NMDA) receptor further autoantibodies against neuronal structures such as the contactin-associated protein-like 2 (CASPR2) were identified, and the important role of CSF in the diagnosis and monitoring of AE arose (1, 2). Additionally, CSF analysis has gained renewed importance in diagnosing multiple sclerosis (MS), particularly with the introduction of the 2017 McDonald criteria (3). These criteria allow the use of CSF oligoclonal bands to demonstrate dissemination in time, facilitating earlier diagnosis and treatment initiation. Despite the growing body of knowledge regarding CSF diagnostics, there remains significant scope to enhance our understanding and improve the diagnostic accuracy for a variety of neurological and psychiatric disorders, including the identification of new biomarkers. This Research Topic covers various topics related to CSF analyses in both neurological and psychiatric diseases.

In the first article, Berger et al. focused on evaluating treatment responses in AE patients, distinguishing between seronegative and seropositive groups. They analyzed data from 150 AE patients treated at their university hospital between 2010 and 2020. Among these, 49 patients did not show specific AE antibodies (seronegative), whereas 57 were identified with known AE antibodies (seropositive). The majority of these patients, approximately 80%, received at least one form of immunotherapy. The authors utilized the modified ranking scale to assess the clinical response to these treatments. A key observation from this study was the comparable effectiveness of immunotherapy in both seropositive and seronegative patients, with no marked differences in treatment outcomes between the two groups. This finding suggests the possibility of unidentified antibodies being present in many of the seronegative AE cases. It also provides valuable insights into managing AE patients, proposing that immunomodulatory therapies could be beneficial for AE treatment regardless of antibody status.

The polyspecific intrathecal immune response (PSIIR), also known as the MRZ reaction (M = measles, R = rubella, Z = zoster), is known to occur in the majority of patients with MS and is considered to have a high diagnostic specificity for this disease (4). In this context, two studies in the Research Topic have explored its diagnostic utility in MS and other conditions. Vlad et al. conducted a study focusing on the differential diagnosis of neurosarcoidosis and MS, a challenging task due to the similarities between these conditions. They retrospectively analyzed CSF samples from 27 neurosarcoidosis patients and compared them with samples from 138 patients with relapsing-remitting MS (RRMS). Notable differences were observed in the CSF profiles of these two groups. Based on their findings, they concluded that the combination of CSF white cell counts >30/μl, absence of CSF-specific oligoclonal bands, and the absence of positive MRZ reaction was able to distinguish between the diseases with a sensitivity and specificity of >92%. In another of the contributing article, Brauchle et al. examined the frequency of PSIIR in older patients (over 50 years) with chronic autoimmune inflammatory neurological diseases other than MS. They analyzed PSIIR test results from 415 patients and found that 76 of them tested positive for PSIIR. Of these, 25 (33%) did not meet the diagnostic criteria for MS or its related spectrum of diseases. Long-term follow-up data was available for 13 patients, all of whom exhibited a progressive course of disease. Based on these observations, Brauchle et al. concluded that PSIIR might serve as a marker for previously unrecognized chronic neurological autoimmune conditions, suggesting a need for further research to better understand this phenomenon.

Subarachnoid hemorrhage (SAH), a potentially life-threatening condition, requires quick diagnosis and treatment. Weller et al. conducted a study exploring the prognostic value CSF metabolite analysis in SAH cases. They focused particularly on the arginine/ornithine ratio in the CSF, investigating its ability to predict cerebral vasospasms and overall clinical outcomes in patients with SAH. Their analysis included 38 SAH patients, and their findings indicated that the arginine/ornithine ratio in the CSF serves as an independent predictor of the clinical outcome. Based on this, they proposed that the arginine/ornithine ratio could be integrated into clinical practice, enhancing the existing prognostic models for SAH.

Finally, Endres et al. detailed an interesting case report of a woman who developed obsessive-compulsive disorder (OCD) post-partum. Their investigation revealed the presence of novel anti-nucleoli autoantibodies in the patient's CSF. These autoantibodies were detected in cerebellar Purkinje cells and cortical neurons, as demonstrated through tissue-based assays using mouse brain slices. This finding suggest an autoimmune link, offering new insights into the origins of such neuropsychiatric conditions.

This second volume of “*New Cerebrospinal Fluid Research to Uncover Mechanisms Driving Neurological and Psychiatric Diseases–Volume II*” in the “Frontiers of Neurology” demonstrates the important role of CSF analysis in diagnosing and predicting neurological and psychiatric diseases. It further emphasizes the need for further research in this field and shows how CSF analysis can directly influence appropriate patient care.

## Author contributions

TS: Supervision, Validation, Writing—original draft, Writing—review & editing. ØT: Methodology, Supervision, Validation, Writing—original draft, Writing—review & editing.

## References

[1] Dürr M Nissen G Sühs KW Schwenkenbecher P Geis C Ringelstein M. German network for research on autoimmune encephalitis. CSF findings in acute NMDAR and LGI1 antibody-associated autoimmune encephalitis. Neurol Neuroimmunol Neuroinflamm. (2021) 8:e1086. 10.1212/NXI.0000000000001086 34697224 PMC8546742

[2] McCracken L Zhang J Greene M Crivaro A Gonzalez J Kamoun M. Improving the antibody-based evaluation of autoimmune encephalitis. Neurol Neuroimmunol Neuroinflamm. (2017) 4:e404. 10.1212/NXI.0000000000000404 29075658 PMC5639462

[3] Thompson AJ Banwell BL Barkhof F Carroll WM Coetzee T Comi G. Diagnosis of multiple sclerosis: 2017 revisions of the McDonald criteria. Lancet Neurol. (2018) 17:162–73. 10.1016/S1474-4422(17)30470-2 29275977

[4] Jarius S Eichhorn P Franciotta D Petereit HF Akman-Demir G Wick M. The MRZ reaction as a highly specific marker of multiple sclerosis: re-evaluation and structured review of the literature. J Neurol. (2017) 264:453–66. 10.1007/s00415-016-8360-4 28005176

